# Effects of progesterone on hippocampal ultrastructure and expression of inflammatory mediators in neonatal rats with hypoxic-ischemic brain injury

**DOI:** 10.3892/etm.2014.1589

**Published:** 2014-02-27

**Authors:** XIAOJUAN LI, JUNHE ZHANG, XIAOQIAN ZHU, RUANLING HOU, XINJUAN LI, XIANHONG DONG, XIAOYIN WANG, CHENGBIAO LU

**Affiliations:** 1 Department of Physiology and Neurobiology, Xinxiang Medical University, Xinxiang, Henan 453003, P.R. China; 2 Department of Biochemistry and Molecular Biology, Xinxiang Medical University, Xinxiang, Henan 453003, P.R. China; 3 Department of Ophthalmology of the Third Affiliated Hospital, Xinxiang Medical University, Xinxiang, Henan 453003, P.R. China

**Keywords:** progesterone, hypoxia-ischemia, brain damage, ultrastructure, tumor necrosis factor-α, nuclear factor-κB

## Abstract

Progesterone (PROG) has been shown to exhibit a protective function against hypoxic-ischemic brain damage. The aim of the present study was to study the effects of PROG in a neonatal rat model of hypoxic-ischemic brain injury. A total of 30 Wistar rats, aged 7 days, were randomly divided into three groups: Sham, model and PROG. The rats in the model and PROG groups underwent a left common carotid artery ligation and were placed in a sealed container at 37°C with 8% O_2_ and 92% N_2_ gas mixtures for 2.5 h to establish animal models of hypoxic-ischemic encephalopathy. The rats in the PROG group were intraperitoneally treated with 8 mg/kg PROG solution 30 min prior to the induction of hypoxia-ischemia. All animals were sacrificed after 24 h and neuronal changes were observed with electron microscopy to investigate the hypoxic-ischemic brain damage. The protein and mRNA expression levels of tumor necrosis factor-α (TNF-α) and nuclear factor-κB (NF-κB) in the hippocampus were detected by immunohistochemistry and quantitative polymerase chain reaction, respectively. The results revealed that the neuronal structures in the sham group were normal. The neuronal structures in the model group exhibited cavitation changes, but these were reduced following PROG administration. The protein and mRNA expression levels of TNF-α and NF-κB in the hippocampal neurons were increased in the model group, and pretreatment with 8 mg/kg PROG was shown to reduce the expression levels of these inflammatory mediators. Therefore, PROG was shown to exert an important protective function in hypoxic-ischemic brain injury by inhibiting the cascade of inflammatory injury induced by TNF-α and NF-κB.

## Introduction

Neonatal hypoxic-ischemic encephalopathy (HIE) is caused by a variety of conditions, including partial or complete hypoxia, cerebral blood flow reduction and suspension-induced neonatal brain injury caused by perinatal asphyxia (1,2). HIE is common during the neonatal period in which the incidence rate is 1–2 per 1,000 cases. It has been reported that 15–20% of children succumb to HIE during the neonatal period and 15–20% of the survivors suffer from permanent neurological deficits, including cerebral palsy and mental retardation (3,4). Although extensive studies on HIE have been performed, an effective therapy remains to be found (5,6).

Inflammation is one of the main causes of hypoxic-ischemic brain damage (7,8), and tumor necrosis factor-α (TNF-α) is one of the most important proinflammatory cytokines. Numerous studies have shown that the expression of TNF-α in the brain rapidly increases following cerebral ischemia (9,10). Activated TNF-α further stimulates the phagocytosis of immune cells and the activated immune cells further stimulate the germination of TNF-α and other substances, including radicals, extracellular matrix proteases, complement factors and cell adhesion molecules. These ultimately induce a variety of biological responses, including tissue damage, shock and apoptosis (11,12).

Ischemia-induced nuclear factor-κB (NF-κB) has a coordinating function in the expression and regulation of proinflammatory genes, which includes responding quickly to a variety of inflammatory stimuli, activating the transcription of a variety of downstream inflammatory genes and holding the central position in the inflammatory response (13,14). NF-κB is the promoter and enhancer of a number of inflammatory mediator genes, including TNF-α, as it contains κB sites that are able to regulate the induction and expression of inflammatory genes (15). This is an important signal for the inflammatory cascade reaction to mediate cerebral ischemia reperfusion. Therefore, inhibiting the cascade reaction of inflammatory injury may reduce cell death, gliosis, edema and apoptosis, indicating that NF-κB has an important function in hypoxic-ischemic brain injury.

Progesterone (PROG) has been shown to protect brain tissues against hypoxic-ischemic brain damage (16–18). Studies on the cerebral protective effects of PROG by Li *et al* demonstrated the effects of PROG in reducing edema following brain injury, which included reducing calcium overload and inhibiting neuronal apoptosis (19–21). Previous studies concerning the protective effects of PROG in the brain have focused on adult rats. Therefore, the present study explored the involvement of TNF-α and NF-κB in the neuroprotective mechanisms of PROG in a neonatal rat model of hypoxic-ischemic brain injury.

## Materials and methods

### Animals and grouping

A total of 30 Wistar rats, aged 7 days and weighing 14.1±2.0 g, were provided by Xinxiang Medical Experimental Animal Center (Xinxiang, China). The rats were randomly divided into three groups with 10 rats in each group. In the sham group, neck incisions were performed without hypoxic-ischemic treatment. In the hypoxic-ischemic (model) group, hypoxic-ischemic treatment was performed, in order to establish animal models. In the drug prevention (PROG) group, the animals were administered 8 mg/kg PROG solution intraperitoneally 30 min prior to the induction of hypoxia-ischemia (21). PROG was purchased from Sigma-Aldrich (batch 0130; St. Louis, MO, USA). The solution was mixed with 0.5 mg/ml sesame oil prior to use. The study was carried out in strict accordance with the recommendations in the Guide for the Care and Use of Laboratory Animals of the National Institutes of Health (Eighth edition, 2011). The animal use protocol was reviewed and approved by the Institutional Animal Care and Use Committee of Xinxiang Medical University (Xinxiang, China).

### Animal model preparation

As described previously (22,23), newborn Wistar rats were anesthetized with 5% isoflurane. The left common carotid artery was isolated and ligated with a silk thread. Following recovery and feeding for 2 h, rats without rotary motion were separated. The remaining rats were placed in a closed container at 37°C with 8% O_2_ and 92% N_2_ introduced at 1.5 l/min for 2.5 h to induce hypoxia-ischemia.

### Ultrastructural changes of the hippocampus in the experimental groups

Following the induction of hypoxia-ischemia for 24 h, the brains of three rats from each group were quickly placed in 2.5% glutaraldehyde at 4°C for 4 h for fixing. The brains were then rinsed with phosphate-buffered saline and fixed again with 1% osmium tetroxide for 1.5 h. Next, the brains were washed with distilled water, dehydrated with a gradient series of ethanol and acetone, embedded in epoxy resin and cut into ultrathin sections. The ultrastructures of the hippocampal neurons were electron stained and then, using a Hitachi H-7500 transmission electron microscope (Hitachi, Ltd., Tokyo, Japan), were observed and images captured.

### Immunohistochemical staining and image analysis to determine TNF-α and NF-κB expression levels in the rat hippocampal tissues of each group

Following the induction of hypoxia-ischemia for 24 h, brain tissues were rapidly obtained and fixed in 4% paraformaldehyde overnight. The tissues were then conventionally dehydrated and embedded in paraffin. The optic chiasm continuous coronal slices were cut into 4-μm pieces, dewaxed, dried and stored at room temperature for immunohistochemical staining of hippocampal TNF-α and NF-κB.

Immunohistochemical techniques were performed using a streptavidin-biotin complex kit, according to the manufacturer’s instructions (Beijing Biosynthesis Biotechnology, Co., Ltd., Beijing, China). Antiphosphate buffer was utilized as a negative control and cells of the cytoplasm and membrane that were stained brown were selected as positive cells. Quantitative analyses of the immunohistochemical reaction products were expressed by mean optical density (MOD), where the MOD values reflected the quantity of products. The results were analyzed using a HMIAS-200 multicolor imaging analysis system (Champion images technology Co., Ltd., Wuhan, China).

Three horizons were randomly selected in the dentate gyrus: CA1, CA2 and CA3 regions. The OD values of the positive stains were measured at a magnification of ×400. The average value was recorded as the MOD value of the hippocampus of the rats.

### mRNA expression levels of TNF-α and NF-κB in the rat hippocampal tissues of each group

Total RNA was extracted from the hippocampus. Absorbance values were determined by UV spectrophotometry (A260/A280, >1.7). Agarose gel electrophoresis revealed three electrophoretic bands (28, 18 and 5 S), indicating that the total RNA extracted had not been degraded and was suitable for use as a template for reverse transcription reactions.

RNA was reverse transcribed into cDNA using a reverse transcription kit (Takara Bio, Inc., Dalian, China), with β-actin serving as an internal reference. The primers were synthesized by Shanghai Sangon Biological Engineering Technology & Services Co., Ltd (Shanghai, China). Primer compositions are shown in Table I. The PCR amplification cycling conditions were as follows: 95°C for 5 min, 94°C for 40 sec, 55°C for 40 sec and 72°C for 30 sec for 30 cycles, and finally 72°C for 5 min. Following 2% agarose gel electrophoresis, the reaction products were observed using a UV analyzer. The electrophoretic bands were photographed and the gray value was obtained through computer imaging analysis. Relative expression levels of the target gene were obtained as follows: (gray value of the target gene zone - gray value of the gel background)/(gray value of the β-actin zone - gray value of the gel background).

### Statistical analysis

Statistical analyses were performed using SPSS software, version 17.0 (SPSS, Inc., Chicago, IL, USA). All data are expressed as mean ± SD. Single-factor analysis of variance was used for comparisons among groups. P<0.05 was considered to indicate a statistically significant difference.

## Results

### Ultrastructural changes in the hippocampus

**Figure 1:**
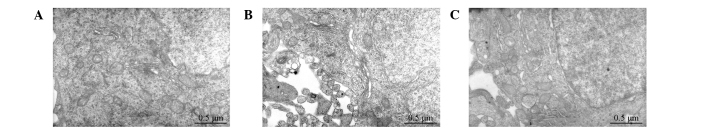
Ultrastructural changes in the hippocampus of the (A) sham, (B) model and (C) PROG groups. PROG, progesterone. Magnification, ×20,000; uranyl acetate and lead nitrate double staining.

In the sham group, the SEM results showed normal neuronal structures, slightly oval neuronal nuclei, evenly distributed nuclear chromatin and normal neutrophils. The mitochondria, rough endoplasmic reticulum, Golgi apparatus and other organelles were visible in the cytoplasm. In the model group, the results revealed cavitation in the neurons, irregular nuclei, cavitation in the nuclear matrix, intracytoplasmic cavitation and swelling of the mitochondrial cytoplasm, fragmenting cristae, cavitation of the cytoplasmic matrix, cavitation changes in the neutrophils and fractured axon neurofilament with dissolution. The neuronal nuclei of the PROG group exhibited oval shapes, evenly distributed nuclear chromatin, occasional cavitation and fracture of the cristae, abundant rough endoplasmic reticulum and mild cavitation changes. The neuronal nuclei of the PROG group were arranged in neat rows (Fig. 1).

### Immunohistochemical staining of TNF-α and NF-κB in the rat hippocampal tissues of each group

**Figure 2:**
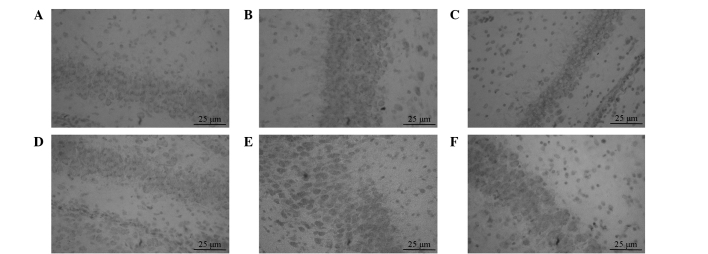
Rat hippocampal tissue expression levels of TNF-α in the (A) sham, (B) model and (C) PROG groups, and NF-κB in the (D) sham, (E) model and (F) PROG groups. TNF-α, tumor necrosis factor-α; NF-κB, nuclear factor-κB; PROG, progesterone. Magnification, ×400; streptavidin-biotin complex (SABC) staining.

Immunohistochemical results showed that the positive expression of TNF-α in the cytoplasm of the hippocampal neurons in the sham group was low, with light staining. The number of positive cells in the model group was significantly increased compared with that in the sham group (P<0.05). However, in the PROG group the number of cells expressing TNF-α was significantly decreased compared with that in the model group (P<0.05). The results are shown in Fig. 2A–C and Table II.

In the sham group, NF-κB expression in the cytoplasm of hippocampal neurons was exhibited in a small number of cells. The number of positive cells in the model group was significantly increased compared with that in the sham group (P<0.05), and NK-κB expression was observed in the cytoplasm and the nucleus. However, in the PROG group the number of positive cells was significantly decreased when compared with that in the model group (P<0.05). The results are shown in Fig. 2D–F and Table II.

Immunohistochemical results indicated that the protein expression levels of TNF-α and NF-κB in the brain tissue increased following hypoxic-ischemic brain damage. However, PROG was capable of reducing the expression levels.

### PROG reduces TNF-α and NF-κB mRNA expression levels in hippocampal tissues

**Figure 3:**
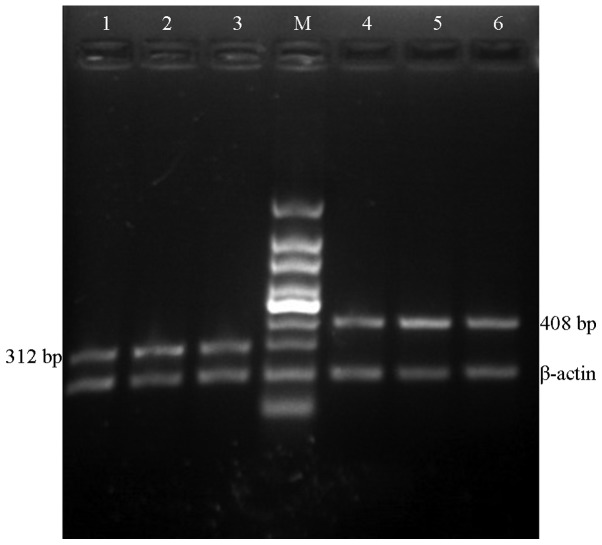
TNF-α and NF-κB mRNA expression levels in the hippocampal tissues of rats. PCR-amplified product bands of TNF-α were 408 bp, bands of NF-κB were 312 bp and bands of β-actin were 198 bp. Lanes 1, 2 and 3: NF-κB expression in the sham, model and PROG groups, respectively. Lanes 4, 5 and 6: TNF-α expression in the sham, model and PROG groups, respectively. TNF-α, tumor necrosis factor-α; NF-κB, nuclear factor-κB; PROG, progesterone; PCR, polymerase chain reaction.

The specific bands of TNF-α and NF-κB mRNA were 408 and 312 bp, respectively. Quantitative polymerase chain reaction (qPCR) results showed that the TNF-α and NF-κB mRNA expression levels, as indicated by the gray values, in the sham group were low. The TNF-α and NF-κB mRNA expression levels in the model group were significantly higher compared with those in the sham group (P<0.05), but were significantly lower in the PROG group compared with those in the model group (P<0.01; Fig. 3 and Table III).

These results indicate that TNF-α and NF-κB mRNA expression levels in brain tissues increase as a result of hypoxic-ischemic brain damage. In addition, PROG exerts a neuroprotective effect by reducing the expression levels.

## Discussion

The results of the present study indicated that the mRNA and protein expression levels of TNF-α and NF-κB in the hippocampus of neonatal rats increased following hypoxia-ischemia for 24 h. However, PROG pretreatment reduced the mRNA and protein expression levels of TNF-α and NF-κB, indicating that PROG reduces the inflammatory response following hypoxic-ischemic injury. Thus, by inhibiting the inflammatory response, PROG exhibits an important protective effect against HIE.

The protective role of PROG on the nervous system has been of significant interest. Chen *et al* (24) demonstrated with a brain injury model that PROG and its reduction products (5α-dihydro-progesterone or allopregnanolone) accumulated around the lesions following injury, indicating that PROG may have a protective effect against brain injury. In brain injury, PROG inhibits the exudation of macrophages and the secretion of TNF-α, interleukin-1 (IL-1) and other cytokines. Specific pathological processes associated with inflammatory factors, including complement factors, nuclear factor (NF) and nitric oxide synthase, are also regulated by PROG (25). The neuroprotective effects of PROG are able to reduce cerebral edema, lipid peroxidation, neuronal death and abnormity, promote stability of the blood-brain barrier and improve cognition following traumatic brain injury and other diseases (26). However, PROG and allopregnanolone reportedly aggravate hypoxic-ischemic brain damage in immature rats (27).

Numerous animal models of neonatal HIE are available. The HIE model prepared by common carotid artery ligation plus hypoxia (8% O_2_ + 92% N_2_, 2.5 h) in the present study is stable and reliable (22,23). Pathological changes mainly manifest as neuronal loss in the lesions. Studies on various animal models with hypoxic-ischemic brain injury have indicated that the hippocampus is the part of the brain most sensitive to ischemic injury (28,29). In the present study, pathological structural changes in the hippocampus of neonatal rats in each group were observed with electron microscopy. The results showed that neuronal structures in the sham group were generally normal, whereas neurons in the model group had cavitation changes due to hypoxic-ischemic neuronal damage. However, hypoxic-ischemic neuronal damage was ameliorated and cavitation reduced following the administration of PROG.

Hypoxic-ischemic brain injury is associated with numerous factors. For example, injury inflammation is one of the main causes of hypoxic-ischemic brain injury (7,8). The upregulation of inflammatory cytokines, chemokines and adhesion molecules constitutes the basis of transforming hypoxic-ischemic injury into inflammatory injury. TNF-α is mainly produced by activated monocytes-macrophages derived from nerve cells, astrocytes, microglia, ependymal cells, vascular endothelial cells and white blood cells in the central nervous system (30). Barone *et al* (31) established permanent and transient cerebral artery occlusion models in spontaneously hypertensive rats. Injecting TNF-α in the forebrain led to a dose-dependent increase in cerebral infarct size and neurological function deficits. By contrast, injecting TNF-α monoclonal antibodies in the brain prior to this reversed the brain damage induced by exogenous TNF-α. However, specific studies have shown that TNF-α exerts a protective effect on brain ischemic tissues. Guo *et al* pretreated rats with transient ischemia of the brain, and found that the expression levels of TNF-α in the brain increased, which significantly reduced ischemic cerebral infarction and the cerebral edema volume following ischemia (32).

NF-κB was identified in 1986 and was termed NF-κB since it specifically binds with κB sequences in the immunoglobulin κ chain gene (33). The expression of NF-κB in the hippocampal cortex and other brain regions of the central nervous system has a wide biological significance, particularly in the process of brain injury and brain degenerative diseases (34). In a resting state, NF-κB combines with its inhibitor, IκB, to form a complex that exists in the cytoplasm and has no biological activity. However, during ischemia and hypoxia, virus infection, mechanical injury, radiation and other stressful conditions, NF-κB is activated and initiates the transcription of involved target genes, resulting in pathophysiological processes, including inflammation, immune response, apoptosis and free radical damage (35,36).

Hypoxic-ischemic injury activates a large number of inflammatory cytokines, including TNF-α and IL-1. These then activate NF-κB, leading to the enhancement of transcription and expression of inflammatory cytokine genes. Thus, NF-κB is a key factor in the regulation of inflammation (37,15).

In the present study, immunohistochemical staining and qPCR results demonstrated that the mRNA and protein expression levels of TNF-α and NF-κB in the hippocampal tissues increased following 24-h hypoxic-ischemic brain injury. PROG pretreatment reduced the expression of inflammatory mediators, which is consistent with previous experimental data (38).

Therefore, evident brain damage emerges following hypoxic-ischemic brain injury for 24 h. The levels of inflammatory mediators in the brain tissues also increase. PROG, as a preventive medication, may relieve inflammation following hypoxic-ischemic brain injury. Therefore, the present study demonstrates the protective effect of PROG on neuronal hypoxic-ischemic brain damage and provides a new method of prevention against HIE among neonates.

## Figures and Tables

**Table I:** Primers used for PCR.

Gene	Primer sequence	Length, bp
β-actin	5′-ACGTGTCATCCGTAAGTAC-3′ 5′-CTGTGGAGCGAGGGCTCAG-3′	198
TNF-α	5′-GCTCCCTCTCATCAGTTCCA-3′ 5′-TGGAAGACTCCTCCCAGGTA-3′	408
NF-κB	5′-GATACCACTAAGACGCACCC-3′ 5′-CGCATTCAAGTCATAGTCCC-3′	312

TNF-α, tumor necrosis factor-α; NF-κB, nuclear factor-κB; PCR, polymerase chain reaction.

**Table II:** MOD values of TNF-α and NF-κB in the hippocampus (mean ± SD).

Group	TNF-α	NF-κB
Sham	0.20±0.02	0.23±0.03
Model	0.53±0.04^a^	0.63±0.04^a^
PROG	0.32±0.03^b^	0.38±0.03^b^

a P<0.05, vs. sham;

b P<0.01, vs. model.

The calculation method of immunohistochemical positive cells was as follows: Three random hippocampal visual fields were observed under a light microscope (magnification, ×400) and digital images were captured and analyzed for staining. From these the MOD values were calculated. MOD, mean optical density; PROG, progsterone; TNF-α, tumor necrosis factor-α; NF-κB, nuclear factor-κB.

**Table III:** Relative TNF-α and NF-κB mRNA levels in the brain tissues of neonatal rats (mean ± SD).

Group	TNF-α	NF-κB
Sham	0.46±0.04	0.48±0.06
Model	0.88±0.05^a^	0.96±0.11^a^
PROG	0.51±0.05^b^	0.56±0.10^b^

a P<0.05, vs. sham;

b P<0.01, vs. model.

Values are expression levels, calculated from gray values, relative to those of β-actin. PROG, progsterone; TNF-α, tumor necrosis factor-α; NF-κB, nuclear factor-κB.
